# Bleaching versus color change resistant adhesive in the discoloration of bracket-bonded tooth surfaces: an in vitro study

**DOI:** 10.1007/s00784-024-05668-5

**Published:** 2024-04-27

**Authors:** Hasan Karadeniz, Sabahat Yazıcıoğlu

**Affiliations:** 1 Giresun Oral and Dental Health Hospital, Giresun, 28200 Türkiye; 2 Faculty of Dentistry, Department of Orthodontics, University of Ondokuz Mayıs, Atakum, Samsun, 55139 Türkiye

**Keywords:** Bleaching, Bracket, Color change resistant adhesive, Discoloration

## Abstract

**Objectives:**

The aim of this study was to compare the effect of office bleaching of teeth bonded with Transbond XT^TM^ (3M Unitek, Monrovia, CA, USA) (TRXT) and the use of color change resistant Orthocem (FGM, Joinville, Brazil) in bracket bonding on coffee-induced enamel discoloration.

**Materials and methods:**

Eighty premolars were distributed in equal numbers (*n* = 20) to group 1 (TRXT + distilled water), group 2 (TRXT + coffee solution), group 3 (TRXT + coffee solution + bleaching), and group 4 (Orthocem + coffee solution). Color was measured using a SpectroShade Micro (MHT, International, Verona, Italy) device at the beginning (T0), after coloring (T1), after bleaching (T1B), and after debonding (T2). ΔE color change values were calculated as T1-T0, T1B-T0 and T2-T0 differences. The conformity of the data to the normal distribution was examined with the Shapiro–Wilk test. Multiple comparisons were made with Tamhane’s T2 test and Tukey’s HSD test using one-way analysis of variance in the comparison of normally distributed data, and multiple comparisons were made with Dunn’s test using the Kruskal–Wallis H test for comparison of non-normally distributed data. The significance level was set at *p* < 0.050.

**Results:**

A statistically significant (*p* < 0.001) difference was found between the T1-T0 and T2-T0 stages for group 1–4 ΔE values. A statistically significant (*p* < 0.001) difference was also found when the T1B-T0 ΔE values of group 3 were compared with the T1-T0 ΔE values of groups 1, 2, and 4.

**Conclusions:**

After coffee-induced enamel discoloration, bleaching of teeth bonded with TRXT produced acceptable color difference of the incisal, middle, and gingival regions of the crown. In teeth bonded with Orthocem, acceptable color difference was seen only in the middle of the crown.

**Clinical relevance:**

The presented study will guide the clinician on how enamel discoloration side effect of fixed orthodontic appliance can reduce.

## Introduction

In orthodontics, a side effect of treatment applications using fixed or removable appliances [1] is discoloration of the teeth [2]. The etiology of these color changes that occur during orthodontic treatment is multifactorial and their intensity is higher when fixed appliances are used rather than removable appliances, because the color of the composite adhesives used in bracket bonding changes [3, 4]. Moreover, the resin tags irreversibly penetrate the enamel structure [5]. This resin absorption in enamel cannot be reversed by debonding and cleaning procedures [6]. Food dyes, ultraviolet light, abrasive substances, and products caused by corrosion of orthodontic appliances also cause tooth discoloration [7–9]. In this case, although it is thought that bleaching will not be feasible due to the presence of brackets [10, 11], studies have shown that bleaching can be applied to teeth with brackets [12–15]. Another method that may be applied to tackle enamel discoloration is the use of orthodontic adhesive resins [16] with increased ability to withstand color changes in bracket bonding.

However, our search of the orthodontic literature revealed no study comparing the effect of bleaching of bracket bonded teeth and that of bonding with discoloration-resistant orthodontic adhesive on enamel discoloration. Transbond XT^TM^ (3M Unitek, Monrovia, CA, USA) (TRXT) is the most commonly used adhesive for bonding brackets [17]. Before using TRXT, TRXT adhesive primer, an unfilled resin, is applied to the acid-etched enamel surface [18]. In contrast, discoloration-resistant orthodontic adhesive Orthocem (FGM, Joinville, Brazil) is noprimer adhesive resin cement. It also has the advantages of simple and reliable bonding procedure with low risk of contaminated bonding. The composition of TRXT consists of bisphenol A diglycidyl ether dimethacrylate, bisphenol A bis (2-hydroxyethyl ether) dimethacrylate, silane-treated quartz and dichlorodimethylsilane reaction product with silica. The composition of Orthocem consists of bisphenol A diglycidyl ether methacrylate, triethylene glicol dimethacrylate, methacrylated phosphate monomer, silane treated silicon dioxide, camphorquinone and sodium fluoride [19]. Therefore, the aim of the present in vitro study was to compare the effect on enamel discoloration of bleaching of TRXT-bonded bracketed teeth with the effect of using a discoloration-resistant orthodontic adhesive in bracket bonding. The null hypothesis of the study was as follows: There is no difference between the effects of TRXT used with office bleaching [Opalescence Boost (Ultradent, South Jordan, UT, ABD)] and those of an adhesive that is resistant to discoloration (Orthocem) on coffee-induced enamel discoloration.

## Materials and methods

The study was designed in line with the modified CONSORT checklist for in vitro studies [20], and was approved by the Ondokuz Mayıs University Clinical Research Ethics Committee (decision number 2022/285). The material of the study consisted of lower or upper first and second premolars extracted for orthodontic treatment. An orthodontic treatment consent form was used and the inclusion criteria for teeth were as listed below:

1. There is no defect or caries on the tooth surfaces.
2. The teeth have not been treated.
3. The teeth belong to patients aged 12–18.

When ∆Eab color change was the primary measurement, sample size was calculated for 95% confidence (1-α) and 95% test power (1-β) to be at least 60 samples for f = 0.626 effect size [21]. However, 80 teeth were included in the study.

The study groups were formed as follows:

Group 1- (control group) - Transbond XT + distilled water.

Group 2- Transbond XT + coffee solution.

Group 3- Transbond XT + coffee solution + opalescence boost.

Group 4- Orthocem + coffee solution.

The teeth were kept in the dark, at room temperature, and in distilled water, which was renewed once a week. Fifty-two upper premolars and 28 lower premolars were randomly distributed into 4 groups, with the number of upper and lower premolar teeth being equal in each group and numbered individually in boxes with lids. The buccal surfaces of all teeth were cleaned with a brush (OptiShine, Kerr, Bioggio, Switzerland) and pumice (Imipomza, Imicryl, Konya, Türkiye) for 10 s at 10,000 rpm, washed with air-water spray, and kept in distilled water until color measurement.

### Initial color measurement

**Fig. 1:**
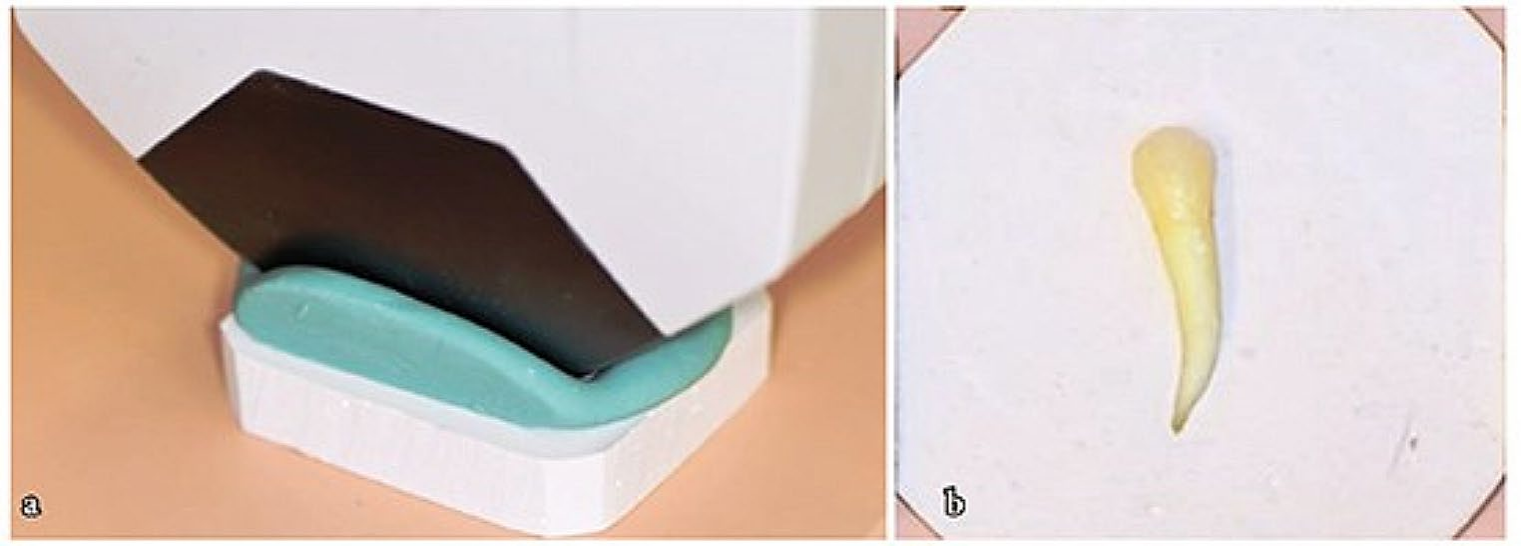
(**a**) Silicone frame for prevent light from penetrating around the teeth (**b**) The tooth embedded in plaster with their buccal surface exposed

It has been reported that color measurements obtained with the SpectroShade Micro (MHT, International, Verona, Italy) device used in the present study are highly reliable and reproducible, and can be used in clinical and experimental studies to determine tooth color and to examine color changes after treatment [22–25]. White and green tiles were used to calibrate the device. Since spectrophotometers can produce variable results under different lighting conditions [26], a silicone frame was made to prevent light from penetrating around the teeth, which were embedded in plaster with their buccal surfaces exposed (Fig. 1).

In the spectrophotometric images, the buccal surface of the tooth was divided into three equal parts, incisal, middle, and gingival, with the program SpectroShade Database Version 1.1.1.0. Data from these three regions based on the CIE L*a*b* system were noted as initial color values (T0). Each measurement was made three times and the averages were calculated. All measurements were obtained by the same researcher (HK) to ensure standardization. After T0 measurements were recorded, the teeth were removed from the plaster and left in distilled water until the coloring stage.

### Bonding the brackets

In groups 1–3, the teeth were treated with 35% gel phosphoric acid (Scotchbond™ Universal Etchant, 3 M Unitek, Monrovia, CA, USA), while in group 4, they were treated with 37% gel phosphoric acid (Condac, FGM), etched for 15 s, washed with water for 15 s, and air dried. MBT 0.022 inch Mini Master Series (American Orthodontics, Sheboygan, NY, USA) metal brackets were bonded to the middle of the buccal surface of the teeth in groups 1–3 using Transbond™ XT Light Cure Adhesive Primer (3 M Unitek, Monrovia, California, USA) and TRXT and in group 4 using Orthocem. Light was applied with a Woodpecker LED-E (Woodpecker Medical Instrument Co., Guilin, China) for 20 s. A 0.014 inch archwire (Adenta GmbH, Gliching, Germany) was ligated to the brackets with elastic (Unistick, American Orthodontics, USA; Power Sticks, Ortho Technology, USA). The teeth were re-immersed in distilled water.

### Coloration stage

Coffee, which releases low polarity yellow pigments potentially penetrating the organic phase in the composite resins and causing coloration [27–29], was used as a colorant in the present study, as in previous studies [30–32]. The solution was prepared with filter coffee (Kuru Kahveci Mehmet Efendi Colombian coffee, Istanbul, Türkiye) at the ratio of 7 g of coffee to 180 milliliters of water.

The teeth were kept at room temperature for 7 days in solutions that were refreshed every 24 h [28]. Then the teeth were removed from the solution and washed with distilled water for 5 s and the brackets were removed with straight bracket removing pliers (Dentaurum; Ispringen, Germany). ARI scores were recorded by evaluating the amount of composite in each tooth and under the bracket under reflector light with the naked eye. After the teeth were re-embedded in plaster without removing the residual adhesive from on the tooth surface, and color measurements were made (T1).

### Bleaching stage

**Fig. 2:**
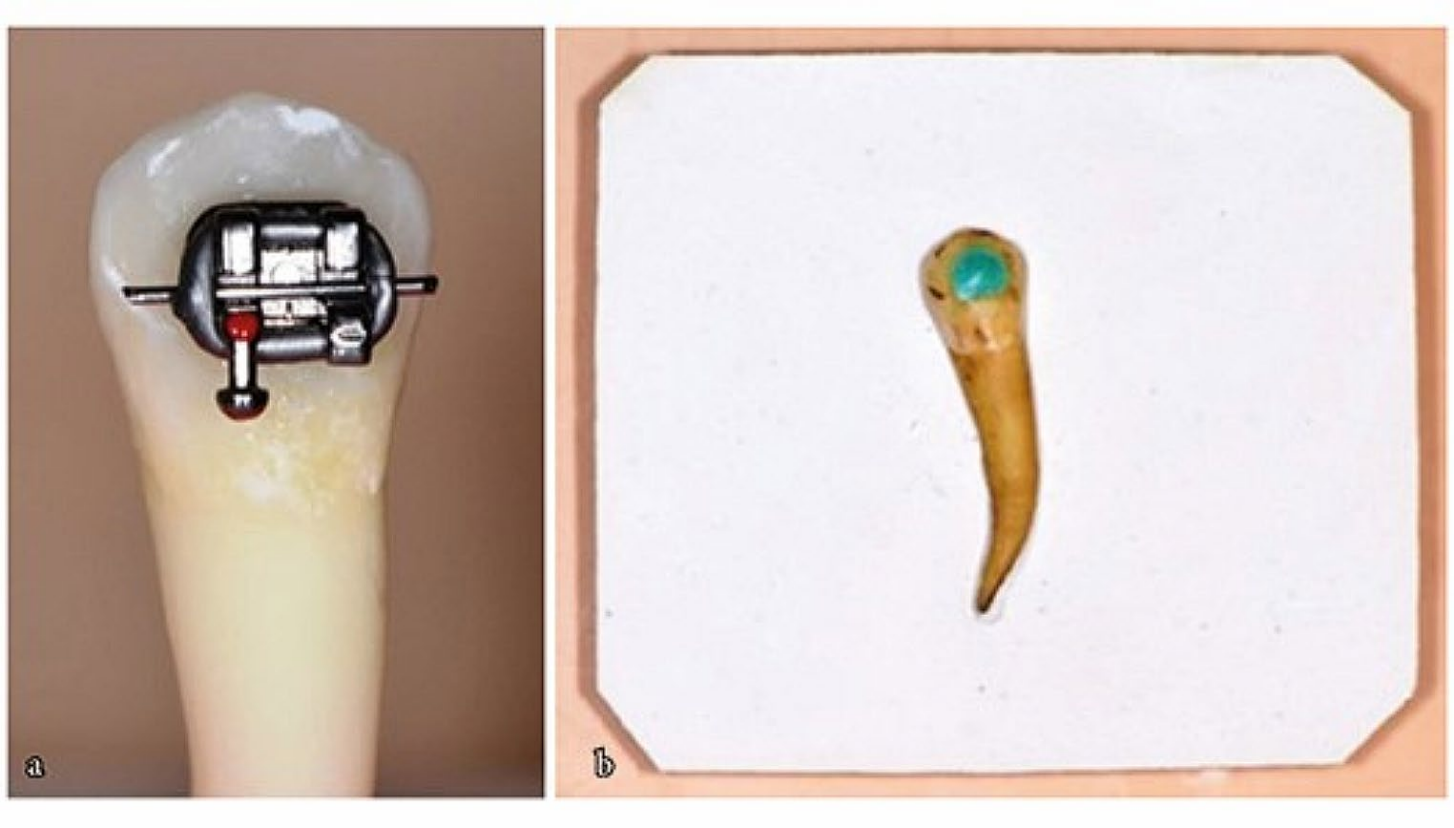
(**a**) Bracket bonded tooth (**b**) Tissue barrier placed in place of the removed bracket

After the T1 measurements of the teeth in group 3, without removing the residual adhesive on the tooth surface a tissue barrier was put in place of the removed brackets and Opalescence Boost was applied to the teeth for 20 min. Then the color measurements (T1B) were repeated (Fig. 2).

### Debonding stage

After completion of T1 and T1B color measurements, residual adhesives on the tooth surfaces were cleaned with a 12-blade tungsten carbide bur (Drendel and Zweiling Diamant GmbH, Kalletal, Germany) at 10,000 rpm and polished for 10 s with pumice in all groups. A new bur was used for every 10 teeth. Then color measurements (T2) were made.

### Calculation of ΔE color change values

As a result of color measurement made with the CIE L*a*b* system, ΔE color change values (T1-T0), (T1B-T0) and (T2-T0) were calculated with the formula [33, 34];

ΔE = [(ΔL*)^2^ + (Δa*)^2^ + (Δb*)^2^]^1/2^.

### Statistical analysis

IBM SPSS V23 was used to analyze the data. The Shapiro–Wilk test was used to test conformity to the normal distribution. Tamhane’s T2 test and Tukey’s HSD test using one-way analysis of variance were used to analyze multiple comparisons when comparing normally distributed data according to groups of three or more. The Kruskal–Wallis H test was used to compare the data that were not normally distributed according to groups of three or more, and multiple comparisons were examined using Dunn’s test. The Mann–Whitney U test was used to compare the data that were not normally distributed according to the paired groups. The results of the analysis were presented as mean ± standard deviation and median (minimum – maximum) for quantitative data. The significance level was set at *p* < 0.050.

## Results

### Intragroup comparisons

A statistically significant difference was determined between the ΔE values of the T1-T0 and T2-T0 stages in groups 1, 2, and 4 for the incisal (*p* < 0.001), middle (*p* < 0.001), and gingival (*p* < 0.001) regions (Table 1).

**Table 1:** Comparison of ΔE values according to tooth regions and stages for Group 1,2 and 4

		ΔE STAGES		
GROUP	REGION	T1-T0	T2-T0	
		median (min. – max.)	median (min. – max.)	**p** ^**c**^
**1**	INCISAL	0.9 (0.1–2.8)^a^	0.2 (0.1–1.0)^b^	**< 0.001**
	MIDDLE	1.2 (0.2–2.7)^a^	0.3 (0.0–2.0)^b^	**< 0.001**
	GINGIVAL	1.4 (0.4 − 3.0)^a^	0.3 (0.0–1.4)^b^	**< 0.001**
	**p** ^**c**^	0.263	0.487	
**2**	INCISAL	17.8 (10.5–22.5)^Aa^	4.0 (1.8–8.0)^Bb^	**< 0.001**
	MIDDLE	8.6 (4.3–16.5)^Bb^	4.4 (1.5–9.5)^Ba^	**< 0.001**
	GINGIVAL	13.5 (4.5–25.6)^Ab^	8.1 (1.0–15.4)^Aa^	**< 0.001**
	**p** ^**c**^	**< 0.001**	**0.006**	
		mean ± sd	mean ± sd	**P** ^**g**^
**4**	INCISAL	12.7 ± 3.1^Df^	4.0 ± 1.3^CDe^	**< 0.001**
	MIDDLE	11.1 ± 2.7^CDf^	3.68 ± 1.3^De^	**< 0.001**
	GINGIVAL	9.2 ± 3.5^Ce^	5.8 ± 2.9^Cd^	**< 0.001**
	**P** ^**g**^	**0.004**	**0.016**	

^c^Kruskall Wallis H test, a-b: There is no statistically significant difference between stages with the same letter (Dunn testi) A-B: There is no statistically significant difference between regions with the same letter (Dunn test) ^g^One-way analysis of variance, d-f: There is no statistically significant difference between stages with the same letter (Tukey’ s HSD test, Tamhane’s T2 test), C-D: There is no statistically significant difference between regions with the same letter (Tukey’ s HSD test, Tamhane’s T2 test) sd: Standard deviation

There was also a statistically significant difference between the ΔE values of the T1-T0, T1B-T0, and T2-T0 stages in group 3 for the incisal (*p* < 0.001), middle (*p* < 0.001), and gingival (*p* < 0.001) regions (Table 2).

**Table 2:** Comparison of ΔE values according to tooth regions and stages for Group 3

REGION	ΔE STAGES						
	T1-T0 median (min. – max.)	T1B-T0 median (min. – max.)		T2-T0 median (min. – max.)			*p* ^d^
INCISAL	16.4 (8.8–24.0)^Ab^	9.2 (2.1–15.3)^Aa^		2.9 (0.9 − 7.2)^c^			**< 0.001**
MIDDLE	8.7 (3.4–18.0)^Bb^	5.0 (1.4–10.6)^Bc^		2.5 (0.1–6.3)^a^			**< 0.001**
GINGIVAL	15.7 (9.4–23)^Ab^	8.7 (0.9–14.0)^Ab^		3 (0.5–8.3)^a^			**< 0.001**
*p* ^d^	**< 0.001**	**0.013**		0.467			

^d^Kruskall Wallis H testi, a-c: There is no statistically significant difference between stages with the same letter (Dunn testi), A-B: There is no statistically significant difference between regions with the same letter (Dunn testi)

### Intergroup comparisons

The incisal ΔE values showed a statistically significant (*p* < 0.001) difference between the groups at the T1-T0 and T2-T0 stages. While the ΔE values of groups 2, 3, and 4 were similar in both stages, group 1 showed the lowest value. At the T1-T0 stage, the order of groups in terms of ΔE values was as follows: group 1 (ΔE = 0.9), group 4 (ΔE = 12.5), group 3 (ΔE = 16.4), and group 2 (ΔE = 17.8). At the T2-T0 stage, the order was group 1 (ΔE = 0.2), group 3 (ΔE = 2.9), group 2 (ΔE = 4.0), and group 4 (ΔE = 4.2). A statistically significant (*p* < 0.001) difference was found between the groups for mid-region ΔE values at the T1-T0 and T2-T0 stages. The order of the groups according to mid-region ΔE values at the T1-T0 stage was group 1 (ΔE = 1.2), group 2 (ΔE = 8.6), group 3 (ΔE = 8.7), and group 4 (ΔE = 10.2). At this stage, the ΔE values of groups 2, 3, and 4 were similar to each other, while group 1 showed the lowest value. At the T2-T0 stage, the order from smallest to largest was group 1 (ΔE = 0.5), group 3 (ΔE = 2.5), group 4 (ΔE = 3.6), and group 2 (ΔE = 4.5). A statistically significant (*p* < 0.001) difference was found between the groups for gingival region ΔE values between the T1-T0 and T2-T0 stages. The order of the groups in terms of gingival ΔE values at the T1-T0 stage was group 1 (ΔE = 1.4), group 4 (ΔE = 9.2), group 2 (ΔE = 14.3), and group 3 (ΔE = 15.9), and group 1 (ΔE = 0.3), group 3 (ΔE = 3), group 4 (ΔE = 5.6), and group 2 (ΔE = 8.1) at the T2-T0 stage (Table 3).

**Table 3:** Comparison of ΔE values between groups according to tooth regions at T1-T0, and T2-T0 stages

REGION		STAGE	ΔE VALUES mean ± sd, median (min. – max.)				
			Group 1	Group 2	Group 3	Group 4	*p*
**INCISAL**		T1-T0	0.9 (0.1–2.8)^b^	17.8 (10.5–22.5)^a^	16.4 (8.8–24.0)^a^	12.5 (6.4–17.5)^a^	**< 0.001** ^**e**^
		T2-T0	0.2 (0.1–1.0)^b^	4.0 (1.8–8.0)^a^	2.9 (0.9 − 7.2)^a^	4.2 (1.3–6.5)^a^	**< 0.001** ^**e**^
**MIDDLE**		T1-T0	1.2 (0.2–2.7)^b^	8.6 (4.3–16.5)^a^	8.7 (3.4–18.0)^a^	10.2 (7.3–17.2)^a^	**< 0.001** ^**e**^
		T2-T0	0.5 ± 0.4^c^	4.5 ± 1.9^b^	2.5 ± 1.5^a^	3.6 ± 1.3^ab^	**< 0.001** ^**d**^
**GINGIVAL**		T1-T0	1.4 ± 0.6^c^	14.3 ± 6.5^b^	15.9 ± 3.6^b^	9.2 ± 3.5^a^	**< 0.001** ^**d**^
		T2-T0	0.3 (0.0–1.4)^c^	8.1 (1.0–15.4)^b^	3 (0.5–8.3)^a^	5.6 (0.9–12.0)^ab^	**< 0.001** ^**e**^

^d^One-way analysis of variance, ^e^Kruskall Wallis H test, a-c: There is no statistically significant difference between groups with the same letter (Tamhane’s T2 test, Dunn test) sd: Standard deviation

A statistically significant difference between the T1B-T0 ΔE values of group 3 and the T1-T0 ΔE values of groups 1, 2, and 4 was seen in the incisal (*p* < 0.001), middle (*p* < 0.001), and gingival (*p* < 0.001) regions (Table 4).

**Table 4:** Comparison of group 3 T1B-T0 ΔE value and group 1,2,4 T1-T0 ΔE values between groups

			ΔE values mean ± sd, median (min. – max.)			
		T1B-T0		T1-T0		
		Group 3	Group 1	Group 2	Group4	*p* ^d^
REGION	**INCISAL**	12.3 ± 5.8^a^	1.1 ± 0.7^c^	17.2 ± 3.7^b^	12.7 ± 3.1^a^	**< 0.001**
	**MIDDLE**	7.3 ± 3.6^b^	1.5 ± 0.6^c^	9.0 ± 2.9^ab^	11.1 ± 2.7^a^	**< 0.001**
	**GINGIVAL**	12.0 ± 5.4^ab^	1.4 ± 0.6^c^	14.3 ± 6.5^b^	9.2 ± 3.5^a^	**< 0.001**

^d^One-way analysis of variance, a-c: There is no statistically significant difference between groups with the same letter (Tamhane’s T2 testi)

sd: Standard deviation

### Comparison of ARI scores between adhesives

A statistically significant difference was found between ARI scores after the removal of 58 brackets bonded with TRXT and 20 brackets bonded with Orthocem (*p* = 0.026). The median ARI value of the TRXT group was 2, while the median ARI value of the Orthocem group was 3.

## Discussion

In the present in vitro study, the enamel color changes seen with the use of Orthocem, an orthodontic adhesive with increased resistance to color changes, were compared with those seen with the use of in-office bleaching on teeth bonded with TRXT. This comparison was based on coffee-induced external discoloration. Reduction of the internal and external discoloration of orthodontic adhesives is necessary for achieving lower enamel discoloration [11].

It has been reported that tooth color changes are observed in particular in the center of the buccal surfaces where brackets are placed [35]. The current study evaluated the buccal surface of the tooth by dividing it into three parts, namely incisal, middle, and gingival, and employed a ΔE threshold value of 3.7. Because ΔE < 1 is clinically not visible color difference, 1 ≤ ΔE ≤ 3.7 represents acceptable color difference, while ΔE > 3.7 represents easily visible color difference [36].

In the TRXT and distilled water group, post-debonding ΔE values showed clinically not visible color difference in the incisal, middle, and gingival regions. In contrast, the ΔE values in the incisal, middle, and gingival regions of the teeth bonded with TRXT and immersed in coffee solution showed easily visible color difference of 4.6, 4.5, and 7.4, respectively, after debonding. Increased color change in the gingival region, irregular gingival enamel prisms, thinner enamel compared to other regions, and anatomical variability of the enamel–cementum junction have been found to be associated with higher absorption and adsorption of coffee in this region [37, 38].

In the group bonded with TRXT and single session bleached with Opalescence Boost, ΔE values were 2.9 in the incisal, 2.5 in the middle, and 3 in the gingival regions and showed acceptable color difference across the entire buccal aspect of the tooth after debonding. Similarly, Jadad et al. [39] performed home bleaching in patients with ongoing orthodontic treatment, 10 days before the end of orthodontic treatment and after the brackets were removed. They reported significant whitening in both groups. Sardarian et al. [40] reported that bleaching can be performed during orthodontic treatment and there is whitening in the area under the bracket. Gomes et al. [41] reported that after hydrogen peroxide bleaching in patients undergoing fixed orthodontic treatment, there was a single color tone on the enamel surface, and the bleaching agent dispersed without being affected by the presence of brackets. The results presented by researchers in case reports are in line with the results obtained in the bleaching group in our study.

In the present study, ΔE values at all three sites after debonding showed clinically not visible color difference in the TRXT-bonded and distilled water group. Trakyalı et al. also reported that no effect of rapid aging on the discoloration of orthodontic bonding systems was observed clinically [42].

After immersion in coffee, the middle region color difference in the Orthocem group was greater than that in the TRXT groups. The ARI score calculated at this stage showed that the amount of residual Orthocem on the enamel surface was significantly higher than in the TRXT group. This suggested that the increase in color difference in the middle region was directly proportional to the amount of residual adhesive. After debonding, acceptable color difference of ΔE 3.6 was observed in the middle region in the Orthocem group, with easily visible color difference of ΔE 4.5 in the TRXT group, and acceptable color difference of ΔE 2.5 in the TRXT and bleaching group. This showed that Orthocem was more advantageous regarding coffee-induced discoloration compared to TRXT used without bleaching, and TRXT when used with office bleaching using Opalescence significantly reduced coffee-induced discoloration of the enamel. Against coffee-induced enamel discoloration, bleaching with Opalescence Boost of teeth bonded with TRXT was more effective than using Orthocem as a bonding adhesive. Lunardi et al. [13] also found significant color differences between enamel surfaces exposed to bleaching agents during orthodontic treatments and enamel surfaces of untreated samples. Moreover, it was reported that the color change of orthodontic composites is affected by many factors such as inorganic filler content, monomer type, and degree of polymerization [43]. Faltermeier et al. [11] reported that 4 different orthodontic adhesives, including TRXT, were sensitive to both internal and external discoloration and were insufficient in terms of color stability. Çörekçi et al. [43] taken the threshold value for ΔE as 3.7 and reported a color change above the clinical threshold value for 6 different orthodontic adhesives, including TRXT. The researchers’ results are directly related to the coloration of the adhesive. In the present study, on the other hand, the discoloration of the tooth surface was evaluated where the material exposed to the colorant was present. However, in the groups in which we used TRXT, ΔE values above the clinical threshold value we obtained for enamel after immersion in coffee were consistent with the results reported by other researchers.

Çörekçi et al. [35] investigated the discoloration of enamel in their clinical study using 4 different adhesives, including TRXT. They reported that color changes ranging from 1.12 to 3.34 ΔE units occurred after orthodontic treatment and that the adhesives had similar effects. In the present study, there was no statistically significant difference between TRXT and Orthocem in terms of their effects on enamel color change after debonding.

Easily visible color difference of ΔE was been reported in other studies examining the color change of enamel according to the CIELab formula and the threshold value of 3.7 ΔE after orthodontic treatment with fixed appliances [44–48]. According to Boncuk et all., both the adhesive system and the resin-removal methods are responsible for this change [44]. Gorucu–Coskuner et all were reported that visible and clinically unacceptable tooth color changes after orthodontic treatment, regardless of the etching and adhesive removal techniques [45]. Karamouzos et al. were concluded that after the orthodontic treatment with fixed appliances and during the first year of retention phase color changes may occur on the enamel surface [46]. Niknam et al. were reported that the combined effect of different bonding adhesives, including TRXT, and resin removal techniques created easily visible color differences of ΔE in enamel in all study groups [47]. Kaya and Bilgiç-Zortuk concluded that when flash-free brackets were used and when polished using carbide bur plus soft flex, there was less color change of the enamel [48]. In the current study, the same adhesive removal and polishing technique was applied in each group. However, in parallel with the results of the researchers, it was determined that TRXT, TRXT with bleaching and Orthocem adhesives caused color change in the enamel.

Kaya et al. [49] reported that acceptable color difference occurred on the enamel surface of the teeth with an average ΔE value of 1.89 after fixed orthodontic treatment in their clinical studies using TRXT. In the current study, clinically not visible color difference was observed in the TRXT group that was not immersed in coffee, and easily visible color difference occurred in the TRXT group that was immersed in coffee. The difference between the two studies was only observed when TRXT was exposed to the colorant.

For perceptibility threshold (PT) and acceptability threshold (AT) used to evaluate the clinical performance of dental materials, CIELAB reported 50:50% PT ΔEab = 1.2, 50:50% AT ΔEab = 2.7 [50]. Accordingly, in the present study, perceptible and unacceptable discoloration of the enamel occurred in all groups, except for the middle crown region of the TRXT with bleaching group. In this region, the ΔE value of 2.5, indicating a perceptible but acceptable color difference of the enamel. According to CIELAB ΔE threshold value of 3.7, there is an acceptable color difference in all three regions of the crown in the TRXT with bleaching group was calculated.

The limitations of the present study are that, due to its in vitro design, enamel discoloration, which is caused many factors such as salivary structure, diet, oral hygiene practices, was examined through the effect of a single colorant and the effect of whitening on the bond strength of the adhesive was not evaluated. Therefore, studies on different bleaching applications and bond strength in in vivo conditions are needed.

## Conclusion

Based on the results of our in vitro study,

- Bleaching of teeth bonded with TRXT produced acceptable color difference of the incisal, middle, and gingival regions of the crown.
- In teeth bonded with Orthocem, acceptable color difference was seen in the middle of the crown, while easily visible color difference was seen in the incisal and gingival regions.

## Data Availability

The datasets used and/or analysed during the current study are available from the corresponding author on request.
